# Continuous Fusion of Motion Data Using an Axis-Angle Rotation Representation with Uniform B-Spline †

**DOI:** 10.3390/s21155004

**Published:** 2021-07-23

**Authors:** Haohao Hu, Johannes Beck, Martin Lauer, Christoph Stiller

**Affiliations:** 1 Institut of Measurement and Control Systems, Karlsruhe Institute of Technology (KIT), Engler-Bunte-Ring 21, 76131 Karlsruhe, Germany; martin.lauer@kit.edu (M.L.); stiller@kit.edu (C.S.); 2 Atlatec GmbH, Haid-und-Neu-Straße 7, 76131 Karlsruhe, Germany; jbeck@atlatec.de

**Keywords:** uniform B-spline, data fusion, ego-motion estimation, axis-angle, Rodrigues’ formula, inertial measurement units (IMU), Simultaneous Localization and Mapping (SLAM)

## Abstract

The fusion of motion data is key in the fields of robotic and automated driving. Most existing approaches are filter-based or pose-graph-based. By using filter-based approaches, parameters should be set very carefully and the motion data can usually only be fused in a time forward direction. Pose-graph-based approaches can fuse data in time forward and backward directions. However, pre-integration is needed by applying measurements from inertial measurement units. Additionally, both approaches only provide discrete fusion results. In this work, we address this problem and present a uniform B-spline-based continuous fusion approach, which can fuse motion measurements from an inertial measurement unit and pose data from other localization systems robustly, accurately and efficiently. In our continuous fusion approach, an axis-angle is applied as our rotation representation method and uniform B-spline as the back-end optimization base. Evaluation results performed on the real world data show that our approach provides accurate, robust and continuous fusion results, which again supports our continuous fusion concept.

## 1. Introduction

In the past years, robotic, micro aerial and automated driving technologies have become more and more popular and are intensively investigated. As the localization system is positioned at the beginning of the whole autonomous pipeline, its robustness and accuracy strongly affect the performance of the rest of the pipeline, such as environment perception, decision making and trajectory planning. Therefore, an accurate and robust localization is a key requirement of these kinds of automated driving/flying systems. In order to achieve this goal, different sensor systems are combined, that is, the Global Navigation Satellite Systems (GNSS), feature-based localization system using cameras or LiDARs, odometer system and inertial measurement units (IMU). These different sensor systems provide different types of measurements with different frequencies: the GNSS provides global position measurements; feature-based localization systems provide pose measurements; the odometer system provides pose difference measurements and IMU sensors provide linear acceleration and angular velocity measurements with a very high frequency. Even though this topic is well studied and many approaches have been developed in the past, it is still challenging to fuse the measurements of different types and frequencies accurately and efficiently with as little as possible loss of information. Nowadays, most famous motion fusion approaches are filter-based or pose-graph-based approaches.

Since the motion of driving/flying platforms is usually limited by some physical motion models, it is straightforward to use a filter-based approach to fuse those measurements making use of a corresponding motion model. Filter-based approaches can run with high frequencies so that they can be easily used in realtime applications. However, not every measurement from highly frequent sensors like IMU can be used due to computation power limitations. This will lose information actively and influence the accuracy of fusion results. Besides that, filter-based approaches usually run in a time forward direction and measurements captured in the future will not affect current fusion results.

In contrast, pose-graph-based approaches can perform the motion fusion problem in time forward and backward directions, with which the fusion results can be further refined. Pose-graph-based approaches can theoretically make use of every sensor measurement. One method is to build a pose graph, which has a number of nodes corresponding to the highest measure frequency in a time window. However, the increase of the number of nodes causes an increase of the number of parameters to be optimized and also the computation complexity. Another way to make use of every measurement is to build the pose graph with a reduced number of nodes in the time window, which also reduces the number of parameters and computation complexity. Then, by fusing the high frequency measurements, the influence of measurements on each node need to be considered by applying extra interpolation techniques. Pose-graph-based approaches usually suffer from this problem, since measurements from different sensor systems do not always have the same time stamps. Additionally, filter-based or pose-graph-based approaches have a big disadvantage: they can only provide results for discrete points in time, which can also be unfriendly for further processing steps such as point cloud accumulating and ground surface estimation, which needs motion estimation with a very high frequency.

**Figure 1:**
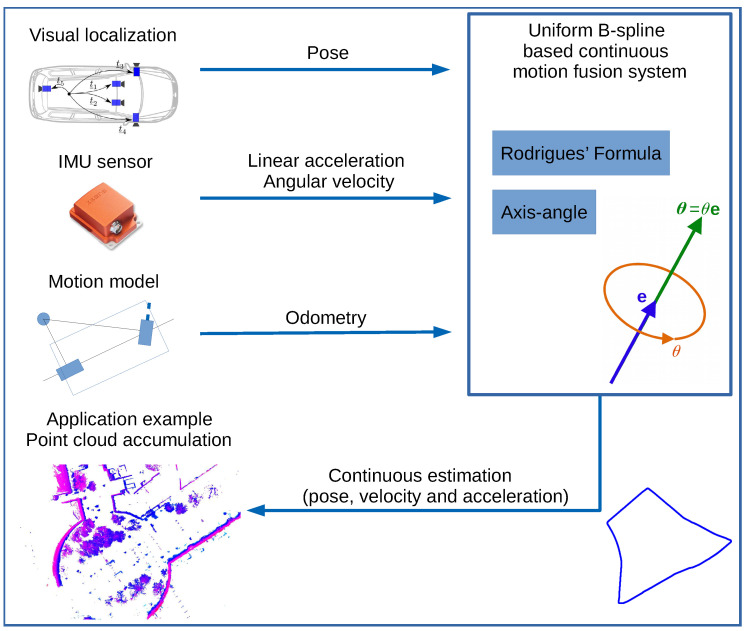
An application example of applying our Uniform B-spline-based continuous motion fusion approach. Our approach can apply pose, velocity and acceleration measurements with different frequencies to optimize two uniform B-splines $S_{t}\;\mathrm{and}\;S_{r}$ (translation and rotation). Afterwards, the fused pose, velocity and acceleration results can be estimated continuously in time.

As discussed previously, filter-based or pose-graph-based approaches have problems by fusing asynchronous motion measurements with different types and frequencies. In this work, we address these problems and present a uniform B-spline-based continuous motion fusion approach, which is shown in Figure 1 and applies asynchronous motion measurements with different types and frequencies and can be optimized in both time forward and backward directions simultaneously. The main contributions of this paper can be summarized as: We present a uniform B-spline-based motion fusion approach, which

- can fuse asynchronous motion measurements with different types (including poses, velocities and accelerations) and frequencies directly without loss of information;
- is processed in the time forward and backward directions to refine fusion results;
- provides pose, velocity and acceleration fusion results continuously in time.

## 2. Related Work

Most existing approaches of fusing motion measurements, including pose, velocity and acceleration, can be divided into two groups: filter-based approaches and pose-graph-based approaches. Filter-based approaches can be further split into two categories: approaches using Kalman Filter (KF) and approaches using Particle Filter (PF).

In [1,2], Extended Kalman Filters (EKF) are used to fuse measurements from visual sensors and IMU to robustly estimate reliable relative robot poses in GNSS denied environments. In the work presented in [3], an EKF-based motion fusion approach is implemented to provide robust localization results for an Unmanned Aerial Vehicle (UAV) combined with a camera mounted on the UAV and a geo-referenced aerial imagery database. The authors of the work [4] believe that a conventional EKF is limited to application in navigation systems by integrating IMU and GNSS sensor systems. The EKF algorithm provides only an approximation to an optimal nonlinear estimation, which may lead to suboptimal performance and sometimes divergence of the filter. Therefore, they use a PF to represent the non-linear and non-Gaussian dynamic models of the IMU sensor. In the work proposed in [5], a robust vehicle self-localization approach in a large scale urban area based on an outdated point cloud map using an IMU and GNSS sensor system is presented. They provide a fusion approach based on a PF, which has successfully performed autonomous vehicle driving in an urban area.

In the above mentioned approaches, the KF or PF algorithms are used to fuse the motion measurements from different sensor systems. They can achieve good results and can be used for realtime localization applications of robots, micro aerial and automated driving vehicles. However, the parameter space in these approaches is usually very large, due to the high dimension of the state vector. In order to achieve robust and accurate fusion results, fine-tuning is needed, which is an overhead. Besides that, filter-based approaches can usually only be optimized in a time forward direction and provide fusion results at discrete points in time.

In the work presented in [6], a graph SLAM based approach is presented for the ego motion fusion of a in-pipe mobile robot, which is designed to work in narrow, dark, wet and dirty environments. In the last ten years, more and more researchers use the pose graph optimization method to solve the ego motion fusion problem for automated driving vehicles equipped with multiple sensor systems. In the works presented in [7,8,9], similar fusion systems are implemented and presented. Firstly, a pose graph is built corresponding to the frequency of odometer or IMU sensor measurements as an initialization of a nonlinear optimization problem. Afterwards, measurements from different sensor systems are added into this nonlinear optimization problem, in which the poses will be optimized as parameters. The frequency of the fusion results depends on the density of nodes in the pose graph. Pose-graph-based approaches can achieve accurate and reliable fusion results and they are widely used for automated driving vehicles. In comparison to filter-based approaches, pose-graph-based approaches can fuse motion measurements in forward and backward directions.

In summary, filter-based approaches work robustly and pose-graph-based approaches use asynchronous measurements with high frequencies. However, both can only provide fusion results at discrete points in time, which may affect the further processing steps and decrease the performance of the whole system. In this work, we present our uniform B-spline-based continuous motion fusion approach by applying the axis-angle rotation representation and Rodrigues’ formula. Our approach fuses linear acceleration and angular velocity measurements from IMU sensors directly without any pre-integrations. It optimizes uniform B-splines in time forward and backward directions, which can refine the fusion results more accurately. Besides that, our approach provides fusion results including pose, velocity and acceleration at unlimited accurate points in time. Additionally, since we only optimize the control points of uniform B-splines, the parameter space is strongly reduced and makes our fusion approach more efficient than others.

## 3. Algorithm Overview

As discussed in Section 1, filter-based and pose-graph-based motion fusion approaches have problems, which are caused by their discrete structures. In order to overcome this problem, we need a time-continuous representation of motion. This representation should have certain properties: continuity in order to be able to model movements and second-order differentiability in order to be able to model velocities and accelerations implicitly. At the same time, this representation must be easily parameterizable and locally-supported in order to avoid unwanted effects. Uniform B-spline satisfies these requirements and we use uniform B-splines to build continuous functions from time *t* to the translation movement T or the rotation movement R. The translation T is modelled as a uniform B-spline $S_{t}$ and the coordinates are used as curve points. The rotation R is modelled as another uniform B-spline $S_{r}$ and the axis-angle coordinates θ are used as curve points. Additionally, the Rodrigues’ formula is applied to compute the rotation matrix R from the axis-angle coordinates θ. Using this rotation representation, the singularity problem can be avoided and it also helps us to estimate rotation, angular velocity and acceleration smoothly and continuously. As we can compute the derivatives of a uniform B-spline, the velocity and acceleration at any time points can be easily estimated.

In this section, firstly, we present our optimization base, uniform B-spline, and discuss the spatial rotation representation methods. Afterwards, we focus analytically on time derivatives of a uniform B-spline. The first and second order time derivatives help us to estimate velocity and acceleration from uniform B-splines. Conversely, the derivation of a uniform B-spline can also be used to build our measurement models. The time derivation and conversion between the axis-angle and rotation matrix are the most important parts and contributions of our fusion approach.

### 3.1. Uniform B-Spline

In order to understand what is a B-spline, we firstly introduce the Bézier curve [10]. Based on the description in [10], a Bézier curve is a weighted sum of n+1 control points, $P_{0},P_{1},\;\ldots ,\;P_{n}$, where the weights are the Bernstein polynomials,

(1) $$X\left(t\right)=\sum_{i=0}^{n}\left(\begin{array}{c} n\\ i \end{array}\right){\left(1-t\right)}^{n-i}t^{i}P_{i},\;0\leq t\leq 1,$$

where $\left(\begin{array}{c} n\\ i \end{array}\right)$ is the binomial coefficients and the variable *t* denotes a range parameter, which is the unique independent variable of the Bézier curve function once the control points are fixed. The Bézier curve of order n+1 (degree *n*) has n+1 control points.

B-splines are a generalization of Bezier curves. In a B-spline, each control point is associated with a basis function. As the basic theory of B-spline is well known, we only provide a brief summary of the concepts and notations. For more details, we refer to the work in [11,12,13,14]. Generally, in comparison with the Bézier curve shown in Equation (1), as shown in [11], a B-spline can be presented with the following function:

(2) $$S\left(t\right)=\sum_{i=0}^{n}B_{i,k}\left(t\right)P_{i},\;t_{\min}\leq t\leq t_{\max},$$

where $P_{0},P_{1},\ldots P_{n}$ are the n+1 control points in total, the $B_{i,k}\left(t\right)$ is the basis function of order k+1(degree *k*) and the *t* is the range parameter. The non-decreasing sequence of real numbers $\left(t_{0},t_{1},\ldots ,t_{m}\right),t_{i}\leq t_{i}+1$ is called the knot vector of the B-spline, where $t_{i}$ is a knot. In this work, to model the motion of an agent, a non-open knot vector is used. As defined in [11], for k=0,1, …, n, the k+1th order (*k*th degree) B-spline basis functions $B_{i,k}\left(t\right)$ in Equation (2) are recursively defined as follows:

(3) $$\begin{array}{cc} B_{i,0}\left(t\right) & =\left\{\begin{array}{cc} 1, & t_{i}\leq t<t_{i+1}\\ 0, & \mathrm{otherwise} \end{array}\right. \end{array}$$

(4) $$\begin{array}{cc} B_{i,k}\left(t\right) & =\frac{\left(t-t_{i}\right)B_{i,k-1}\left(t\right)}{t_{i+k-1}-t_{i}}+\frac{\left(t_{i+k}-t\right)B_{i+1,k-1}\left(t\right)}{t_{i+k}-t_{i+1}}. \end{array}$$

From Equations (3) and (4), it is clear that the knot vector can be any non-decreasing sequence of numbers. We call a B-spline a uniform B-spline, if the knots are equally spaced: $t_{i+1}$−$t_{i}$ = constant. Using such equally spaced knot vectors makes the computation much more efficient, as the basis functions can be precomputed, which makes our motion fusion approach computationally efficient.

The uniform B-spline basis functions share most properties with the B-spline basis functions, such as non-negativity, local support, partition of unity, order of continuity, linear independence and symmetry. These features of a uniform B-spline are also the reasons why we use it as our optimization base for motion fusion. The order of continuity of uniform B-splines makes it possible to estimate the velocity and acceleration, when we present a trajectory with a cubic uniform B-spline. In order to avoid unwanted effects, the uniform B-spline benefits from its local support character. The linear independence allows us to model the translation or rotation movement in 3D space with three variables in one uniform B-spline.

In this work, we use uniform B-splines to fuse linear acceleration and angular velocity measurements from IMU sensors, for which the first and second derivatives of a uniform B-spline are required. From Equation (2), we can see that if we want to derive the B-spline S(t), we only need to compute the derivatives of the basis functions $B_{i,k}\left(t\right)$, since the control points are given as constants. As the basis functions are defined recursively, the time derivatives of the basis functions also need to be computed recursively. The computing of the first and second order time derivatives of the basis functions can be summarized in the following two equations:

(5) $$\begin{array}{cc} \frac{dB_{i,k}\left(t\right)}{dt} & =\frac{dB_{i,k-1}\left(t\right)}{(t_{i+k-1}-t_{i})dt}-\frac{dB_{i+1,k-1}\left(t\right)}{(t_{i+k}-t_{i+1})dt} \end{array}$$

(6) $$\begin{array}{cc} \frac{d^{2}B_{i,k}\left(t\right)}{dt} & =\frac{d^{2}B_{i,k-1}\left(t\right)}{\left(t_{i+k-1}-t_{i}\right)dt^{2}}-\frac{d^{2}B_{i+1,k-1}\left(t\right)}{\left(t_{i+k}-t_{i+1}\right)dt^{2}}. \end{array}$$

We also need to notice that the order (degree) of the used uniform B-spline in this work needs to be selected properly. In order to make the fusion of acceleration measurements possible, the used uniform B-spline should be second order time derivable. To satisfy this requirement, in this work, we chose the uniform cubic B-spline to represent the motion of an agent, which means the parameter *k* in Equations (5) and (6) is 3.

### 3.2. Rotation Parametrization

For the rotation modelling, there are four different parametrization methods: Euler angles, quaternion, rotation matrix and axis-angles. By applying Euler angles, a rotation movement is described using three rotation angles α,βandγ around the x-, y- and z-axis. One disadvantage is that a rotation movement cannot be uniquely defined using three Euler angles, which is caused by the called “gimbal lock” effect and makes this representation method unsuitable for our application.

The methods quaternion q and rotation matrix R are also not suitable for our approach since they are not a minimal parameterization of a rotation movement. A quaternion q in 3D space is a vector with four elements and a rotation matrix R is a 3 × 3 matrix and consists of nine elements. The quaternion vector q describes a rotation movement, only if it is normalized and the matrix R describes a rotation, only if the condition $R^{-1}=R^{T}$ is satisfied. If we use these two methods to represent a rotation movement, some hard conditions need to be considered, which we actually want to avoid in our approach. A rotation matrix R cannot describe unique Euler rotate angles. For example, once the conventions are fixed for Euler angles, the rotation matrix R for the rotate angles α,βandγ is the same for the new rotation angles α+2π,β+2πandγ+2π. The mapping from the rotation movements to the rotation matrix is period instead of unique. For these reasons, we decide to use the axis-angle as the rotation parametrization method.

The axis-angle representation $\theta =\theta \bar{e}$ of a rotation movement parametrizes a rotation in a three-dimensional Euclidean space by two quantities: a unit vector $\bar{e}$ indicating the direction of the axis of rotation, and an angle θ describing the magnitude of the rotation about this axis. Each axis-angle coordinate θ represents a rotation and every rotation has a unique axis-angle representation. A continuous rotation movement is also continuous in the axis-angle space, which allows us to achieve a very good approximation of the movement in the real world using axis-angle representation and continuous uniform B-splines. Additionally, by using the Rodrigues’ rotation formula, a unique rotation movement can be easily determined from each axis-angle vector. Based on the time derivatives of a uniform B-spline using the axis-angle representation, the angular velocity and acceleration can also be estimated. The Rodrigues’ formula provides us an algorithm to compute the exponential map from so(3), the Lie algebra of SO(3) to SO(3), without actually computing the full matrix exponential:

(7) $$R=I_{d}+\sin\theta {\left[\bar{e}\right]}_{\times }+\left(1-\cos\theta \right){\left[\bar{e}\right]}_{\times }^{2},$$

where

(8) $${\left[a\right]}_{\times }:=\left(\begin{array}{ccc} 0 & -a_{3} & a_{2}\\ a_{3} & 0 & -a_{1}\\ -a_{2} & a_{1} & 0 \end{array}\right)\in {\mathrm{Skew}}_{3}$$

is the skew-symmetric matrix of the vector a such that ${\left[a\right]}_{\times }b=a\times b$, for all $a={\left(a_{1},a_{2},a_{3}\right)}^{T}$, $b\in R^{3}$. The $I_{d}$ represents the identity matrix. The axis-angle coordinates $\theta :=\theta \bar{e}$ are a natural and compact rotation representation in terms of its geometric building blocks [15]. The Rodrigues’ formula Equation (7) is a closed form expression of the exponential map [15],

(9) $$R=\exp\left({\left[\theta \right]}_{\times }\right):=\sum_{k}=0^{\infty }\frac{1}{k!}{\left[\theta \right]}_{\times }^{k}=\sum_{k}=0^{\infty }\frac{\theta^{k}}{k!}{\left[\bar{e}\right]}_{\times }^{k}.$$

Moreover, the axis-angle coordinate θ can be used either locally or globally [16], which makes it possible to describe global trajectories. More observations of this rotation parametrization method can be found in the work proposed in [17]. Therefore, based on this analysis, we use the axis-angle representation to model the rotational motion.

### 3.3. Time Derivative of a Rotation

As described in Section 3.1, in order to estimate velocity and acceleration from a uniform B-spline presenting poses, not only the derivative of the uniform B-spline itself but also the time derivatives of the poses are required. Considering the translation part, the linear velocity can be estimated using v=dT/dt and the linear acceleration can be estimated using $a=d^{2}T/dt^{2}$ from the translation coordinate vector $T={\left(x,y,z\right)}^{T}$.

In contrast to the simple estimation of the linear velocity v and acceleration a, the estimation of the angular velocity ω and acceleration α from a rotation movement w.r.t. the axis-angle coordinates is more complex and computation unfriendly. Although formulas exist to express the derivative of the exponential map in general Lie groups [18,19], in this paper, we use the analytical formula presented in [20], which computes the derivatives of the rotation matrix itself w.r.t. the axis-angle coordinates in a simple way. The derivative of $R\left(\theta \right)=\exp({\left[\theta \right]}_{\times })$ described in Equation (9) w.r.t. its axis-angle coordinates $\theta ={\left(\theta_{1},\theta_{2},\theta_{3}\right)}^{T}$ can be formulated as follows:

(10) $$\frac{\partial R}{\partial \theta_{i}}=\frac{\theta_{i}{\left[\theta \right]}_{\times }+{\left[\theta \times \left(I_{d}-R\right)b_{i}\right]}_{\times }}{{∥\theta ∥}^{2}}R,$$

where $b_{i}$ denotes the i-th vector of the standard basis in $R^{3}$ and $I_{d}$ is a 3 × 3 identity matrix. Considering the case θ→0, Equation (10) cannot be used directly. Instead, we need to do some approximations. As presented in [20], the derivative at the identity can be shown by computing the limit as θ→0 of Equation (10). It makes use of the facts that $\lim_{\theta \to 0}R=I_{d}$ and $\lim_{\theta \to 0}\left(I_{d}-R\right)/∥\theta ∥=-{\left[\bar{e}\right]}_{\times }$

(11) $$\begin{array}{c} \begin{array}{cc} \lim_{\theta \to 0}\frac{\partial R}{\partial \theta_{i}}\; & =\lim_{\theta \to 0}\left(\frac{{\bar{\theta }}_{i}{\left[\bar{e}\right]}_{\times }+{\left[\bar{e}\times \left(I_{d}-R\right)b_{i}\right]}_{\times }}{{∥\theta ∥}^{2}}R\right)\\ \; & ={\bar{\theta }}_{i}{\left[\bar{e}\right]}_{\times }-{\left[\bar{e}\times \left({\left[\bar{e}\right]}_{\times }b_{i}\right)\right]}_{\times }\\ \; & ={\left[{\bar{\theta }}_{i}\bar{e}-{\left[\bar{e}\right]}_{\times }^{2}b_{i}\right]}_{\times }\\ \; & ={\left[b_{i}\right]}_{\times }. \end{array} \end{array}$$

More details on Equations (10) and (11) can be found in [20]. As described in Section 1, we use uniform B-splines to present the movement of an agent. By applying the axis-angle coordinate θ as the curve point of the uniform B-spline, this uniform B-spline can be used to describe the rotational motion: S(t)=θ(t). At the same time, the control points of this uniform B-spline also use the axis-angle coordinate representation: $P_{i}=P_{\theta }$. Using the axis-angle coordinate θ and the uniform B-spline representation shown in Equation (2), the first and second order time derivatives of the curve points S(t)(axis-angle coordinate θ) can be calculated from the rotation uniform B-spline $S_{r}$, which are used, combined with Equations (5) and (6), to estimate the velocity and acceleration:

(12) $$\begin{array}{cc} \frac{dS}{dt} & =\sum_{i=0}^{n}\frac{dB_{i,k}\left(t\right)}{dt}P_{i} \end{array}$$

(13) $$\begin{array}{cc} \frac{d^{2}S}{dt^{2}} & =\sum_{i=0}^{n}\frac{d^{2}B_{i,k}\left(t\right)}{dt^{2}}P_{i}, \end{array}$$

where $P_{i}$ are the control points of the uniform B-spline. Additionally, the relationship between the first order derivative of the rotation matrix R and the skew matrix of the angular velocity ω can be presented in equation

(14) $${\left[\omega \right]}_{\times }=\frac{dR}{dt}R^{-1}=\frac{dR}{dt}R^{T},$$

with which we can easily compute the angular velocity ω from the rotation matrix R and the first order derivative dR/dt. If it is necessary and useful, we can also calculate the angular acceleration α from R, dR/dt and $d^{2}R/dt^{2}$ using the equation below:

(15) $${\left[\alpha \right]}_{\times }=\frac{d^{2}R}{dt^{2}}R^{T}+\frac{dR}{dt}{\left(\frac{dR}{dt}\right)}^{T}.$$

Since we do not use any sensors in this work, with which we can directly measure the angular acceleration α, we do not need Equation (15) to build a corresponding measurement model for the angular acceleration measurements. However, it can be helpful for other optimization applications, in which the angular accelerations α are also considered as measurements. For example, by trajectory planning for an automated driving vehicle, we also need to consider the angular acceleration to make the rotation motion of the vehicle as smooth as possible. Combining with Equation (12), afterwards, the angular velocity ω is carried out using a uniform B-spline $S_{r}$ with the axis-angle coordinates θ:

(16) $$\begin{array}{c} {\left[\omega \right]}_{\times }=\frac{dR}{dt}R^{T}=\left[\frac{\partial R}{\partial \theta_{1}},\frac{\partial R}{\partial \theta_{2}},\frac{\partial R}{\partial \theta_{3}}\right]\frac{d\theta }{dt}R^{T}, \end{array}$$

which can be used combining with Equations (9)–(14) to compute the angular velocity ω. Now, the angular velocity ω is derived as a function of the rotation matrix R and the axis-angle coordinates θ, which can be easily estimated from the uniform B-spline $S_{r}$. As the main contribution of our fusion approach, Equation (16) is used as the measurement model for angular velocity measurements from an IMU sensor.

### 3.4. Motion Model

In this paper, we evaluate our fusion approach on the data collected from our experimental vehicle *Bertha One* [21] equipped with a multiple camera setup, LiDAR system, an IMU sensor and the car interface speed odometer. In order to fuse the odometer measurements directly, we apply a single bicycle track model, which can be described during optimization as a cost residual with:

(17) $$res_{\mathrm{motion}}=\int_{t_{1}}^{t_{2}}\dot{\psi }-v_{r}\cdot \frac{\tan\delta }{l}\;dt,$$

where the $t_{1}$ and $t_{2}$ describe the time interval to be considered. The $\dot{\psi }$ presents the roll rate and $v_{r}$ the rear axle velocity of the vehicle. The δ is the steering angle, which can be easily obtained from the vehicle odometry sensor. The constant *l* is the distance between the rear and front axles of the experimental vehicle. During the optimization process, the squared form of this residual term from Equation (17) is minimized.

### 3.5. Uniform B-Spline-Based Fusion System Concept

After presenting the uniform B-spline theory, axis-angle rotation representation, Rodrigues’ Formula and time derivatives of rotations, we present our novel continuous motion fusion approach in a system view manner. In order to model the movement of an agent, we use two uniform B-splines $S_{t}$ and $S_{r}$ to describe the translational and the rotational motion separately. Both these uniform B-splines present a function from the independent variable time *t* to the corresponding definite variable motion: T or R. Like pose-graph-based approaches, the core of our uniform B-spline-based approach is to solve a nonlinear optimization problem. In this optimization problem, the control points of the uniform B-splines are the parameters to optimize and that means that after the optimization process we obtain the optimized control points/ the optimized uniform B-splines. After these two uniform B-splines $S_{t}$ and $S_{r}$ are optimized, the motion of the agent at every time point on the curve could be easily estimated. The measurements from different sensors are used to build the error residuals by applying the corresponding measurement models. In order to present the translational motion of an agent, we use a uniform B-spline $S_{t}$, in which the control points are parametrized directly using the translation coordinate vector $T={\left(x,y,z\right)}^{T}$. For the translational motion, the translation linear velocity measurements from the visual localization system and linear acceleration measurements from the IMU sensor can be applied. For that, the time derivatives of a uniform B-spline using Equations (3)–(6) presented in Section 3.1, combined with the time derivatives of the translation coordinate vector $T={\left(x,y,z\right)}^{T}$ itself, are used as measurement models. To present the rotational motion of the agent, we use another uniform B-spline $S_{r}$, in which the control points are parametrized with the axis angle rotation vector θ. For the rotational motion of the agent, the rotation measurements from the visual localization system and angular velocity measurements from the IMU sensor can be used. In comparison with the measurement models used for the estimation of the translational uniform B-spline $S_{t}$, the measurement models for the rotational uniform B-spline are more complicated. Excepting the time derivatives of a uniform B-spline itself, the first and second order time derivatives of a rotation motion (the angular velocity and acceleration) using the axis angle rotation representation need to be applied to fuse the different rotation measurements, which are presented in Equations (14)–(16) in Section 3.3. Based on the dynamic characteristics of a driving vehicle, we chose a reasonable and meaningful control point density: ten control points per second. The pose measurements coming from the visual localization system can be used directly to build residuals with the pose difference, due to the simple measurement model. However, the linear acceleration a and angular velocity ω measurements from an IMU sensor cannot be used directly and the needed measurement models are described in Section 3.3. In the case of vehicles, a single bicycle track motion model can be also modeled as residuals to integrate odometer measurements. During the optimization process, measurements from different sources are weighted based on the estimation uncertainty.

## 4. Experimental Evaluation

In this section, we evaluate our uniform B-spline-based motion fusion approach with a real world dataset. For evaluation, first we fused the collected sensor motion measurements using our uniform B-spline-based approach and a baseline approach: the pose-graph-based approach. As described in Section 3, our uniform B-spline-based approach can directly handle the IMU measurements and, for the pose-graph-based baseline approach, we implemented an IMU pre-integration using the method proposed in [22] to initialize the pose graph. Then, we compared the fused results from both those approaches in two ways: fusion accuracy and runtime analysis, with which we can show why our uniform B-spline-based motion fusion approach outperforms the pose-graph-based approaches. Afterwards, we evaluated the pose, velocity and acceleration estimation of the fused results using our uniform B-spline-based fusion approach at the time stamps, where the motion information is estimated by the visual localization system explicitly. Additionally, we plotted the fused results, including pose, velocity and acceleration, using our approach to show how realistic and accurate the fused results are, fitted to the movement of an experimental driving vehicle. In order to show the advantages of time-continuous estimation of the motion state using our motion fusion approach, we used the fused results to accumulate the high frequency LiDAR point cloud raw packets, which were also correctly stamped. For comparison, we used the fused results using the pose-graph-based approach or the directly linear interpolation of the visual localization vehicle poses for point cloud accumulation.

### 4.1. Sensor Setup

The real world data for evaluation were collected from our experimental vehicle equipped with a multiple camera setup behind the windshield, 4 Velodyne VLP-16 Puck LiDAR sensors, a car interface speed odometer sensor and an Xsens IMU sensor. The multiple camera setup was intrinsically and extrinsically calibrated using the approach presented in [23] with checkerboards. In order to calibrate the LiDAR sensors, the multiple camera setup and our vehicle rear axis coordinate system extrinsically, we applied the methods proposed in [24,25]. The extrinsic calibration between the Xsens IMU sensor and the vehicle rear coordinate system was coarsely initialized and jointly optimized within this work to develop a uniform B-splines-based motion fusion approach together.

### 4.2. Data Set

**Figure 2:**
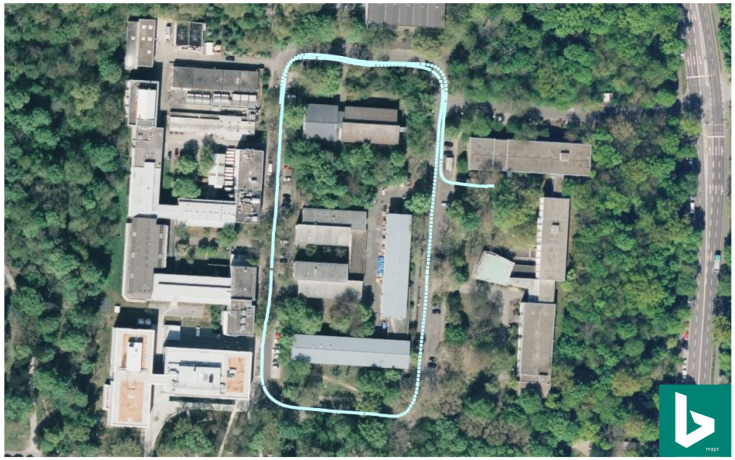
The vehicle driving track used for evaluation shown in Bing aerial imagery using the GNSS measurements for visualization.

For the evaluation of our uniform B-spline-based motion fusion approach, a small track was recorded using our experimental vehicle with the previously described sensor setup. In order to show this track more clearly, as shown in Figure 2, we plotted this track in Bing aerial imagery using the GNSS measurements of the recording. In this evaluation dataset, there was a total of 613 GNSS global position measurements, 1296 multi-view image frames, 12,352 car interface speed odometer measurements and 12,348 Xsens IMU linear acceleration and angular velocity measurements. By applying the surround view visual mapping approach proposed in [26], we built a visual mapping and localization system with the multiple camera setup, from which we can estimate the vehicle poses using the camera images. In this work, the GNSS measurements were only used to visualize the track in the Bing aerial imagery and also to coarsely geo-align our visual localization trajectory. From the Xsens IMU sensor, we received linear acceleration and angular velocity measurements with a high frequency of 100 Hz. Besides that, we also obtained motion states measurements from our car interface speed odometer sensor with a frequency of 100 Hz. It should be noted that these measurements were captured with the same time reference supported by a triggering hardware, but asynchronously.

### 4.3. IMU Extrinsic Calibration

In order to fuse the different motion measurements from different sensors with different frequencies, the introduced sensor systems should first be intrinsically and extrinsically calibrated together. As mentioned previously, we assumed that our multiple camera setup and the LiDAR sensors were already intrinsically and extrinsically calibrated to the vehicle rear axis coordinate system. To recalibrate the IMU sensor to our vehicle rear axis coordinate system, we added the spatial transformation between the IMU sensor and the vehicle rear axis coordinate system as parameters into the fusion optimization problem initialized from a manually measured meaningful coarse calibration. Since the spatial transformation between the IMU sensor and our vehicle rear axis is usually unchanged without any hardware modification, we only needed to estimate the extrinsic calibration parameters once. After the optimization problem was solved and the extrinsic calibration parameters were jointly estimated, we kept hold of the extrinsic calibration parameters between the Xsens IMU sensor and the vehicle rear axle coordinate system for the further usage of our motion fusion approach.

### 4.4. Uniform B-Spline vs. Pose Graph Optimization

**Figure 3:**
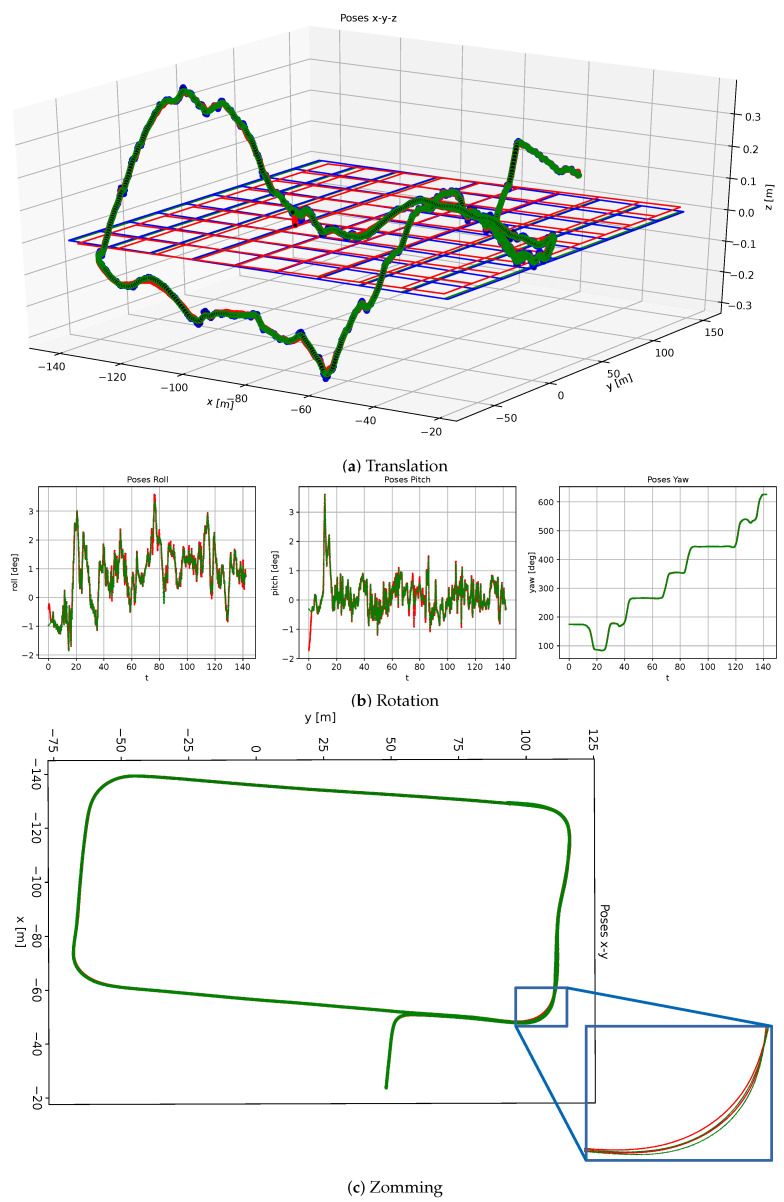
Plotting of the visual localization poses (highlighted with blue color), motion fusion results using pose-graph-based approach and IMU pre-integration (highlighted with red color), and using our uniform B-spline-based approach (highlighted with green color) for comparison. (**a**) The 3D position plotting. (**b**) The 3D orientation plotting with roll, pitch, and yaw angles. (**c**) The 2D position plotting with detailed zooming of one critical part. Here, we can see that the green curve is closer to the blue curve than the red curve, which means that our approach can estimate the motion fusion results more accurately.

In order to show the advantages of our uniform B-spline-based motion fusion approach in comparison to the pose-graph-based approach, we evaluated both approaches on our evaluation dataset. As described previously, the IMU linear acceleration and angular velocity measurements were directly applied by our approach. In contrast to that, for the pose-graph-based approach, we first pre-integrated the IMU measurements for the initialization of the pose graph. The results are shown in Figure 3 with the original visual localization poses in blue, the fused motion results using the pose-graph-based approach in red, and the fused motion results using our uniform B-spline-based approach in green. From the 3D position plotting in Figure 3a and the 3D orientation plotting using Euler angles in Figure 3b, we can see that both approaches can basically provide good motion fusion results, which presents the reasonable driving movement of a vehicle. However, from the 2D position plotting shown in Figure 3c, it is also clear that the red curve differs from the blue curve in the zoomed critical area. This means that there are some drifts in the fused motion results using the pose-graph-based approach. In comparison to that, our motion fusion approach provides more accurate results. The reason for this could be the accumulated drift errors during IMU data pre-integration.

### 4.5. Runtime Analysis

By analyzing the evaluation results in Section 4.4, we know that, although some drifts exist, the fused results using the pose-graph-based approach are basically as good as the results using our uniform B-spline-based approach. However, to obtain these similar results, the runtime of the fusion process by using these two different motion fusion approaches is very different. The pose-graph-based fusion process on our evaluation track, which has a driving length ofabout 140 s, takes about 10 s running on a single Intel CORE i7 7th Gen CPU. In contrast to that, the fusion process using our uniform B-spline-based approach takes less than 1s, which shows a big advantage for online realtime processing applications. The main technology of these two fusion approaches is a non-linear optimization, and the two main factors that influence the runtime performance are: the number of parameters to be optimized and the number of measurements. The number of measurements remains unchanged for both approaches. However, as described in Section 3, the pose-graph-based approach optimizes the pose-graph directly, which is built based on the IMU measurement frequency (in our evaluation dataset: 100 Hz). That means the number of parameters to be optimized is 12,348 × 6 = 74,008 for 12,348 6d poses. In contrast to that, with a meaningful selection of control point density of 10 per second, the number of parameters by using our uniform B-spline-based approach is only 120×10×6=7200. The strongly reduced parameter number makes our approach much faster than the pose-graph-based approach. From this runtime analysis, for the motion fusion in a time horizon of about 10 s, our motion fusion approach needs less than 100 ms, which shows the realtime capability of our approach. Actually, for online applications, it is usually enough to fuse data in a time horizon of 3–5 s instead of 10 s. This shows that the performance of our approach is more than realtime capable.

### 4.6. Pose, Velocity and Acceleration of the Fused Results

**Figure 4:**
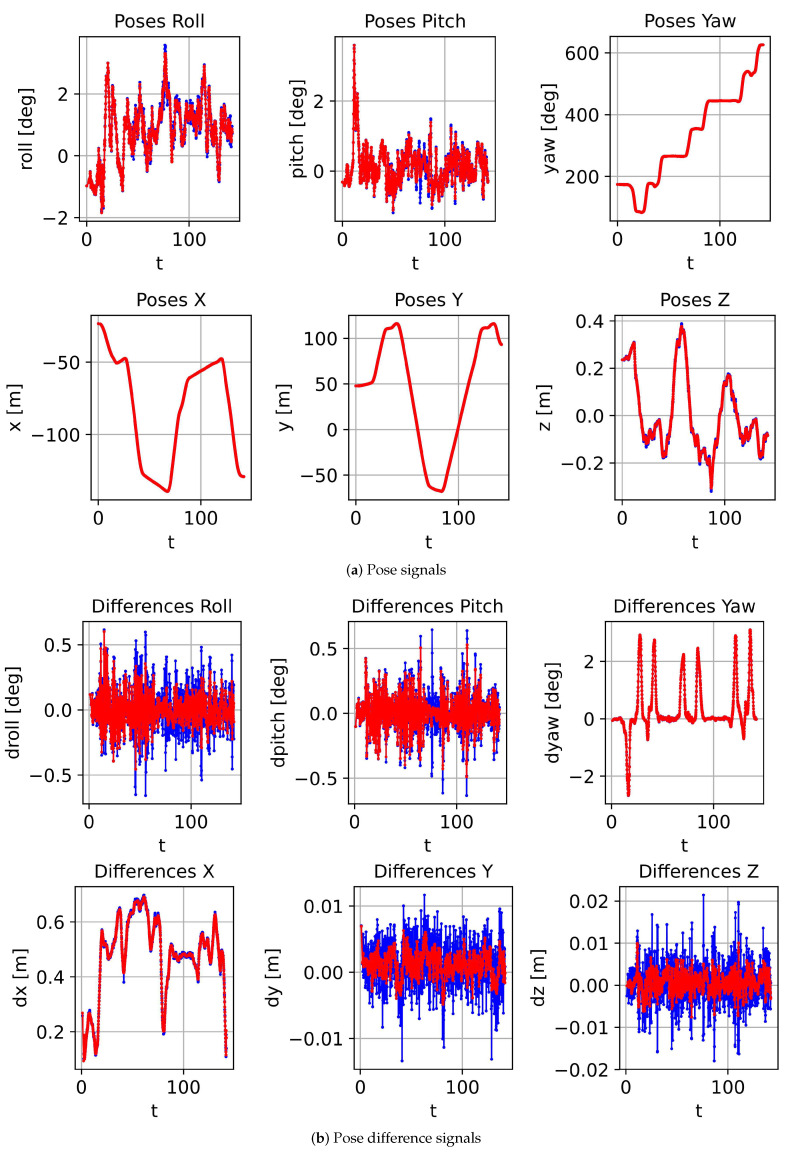
Pose signals and pose difference signal details of the visual localization results (highlighted with blue color) and the fused results by applying our uniform B-spline-based motion fusion approach (highlighted with red color). (**a**) The pose signals. (**b**) The pose difference signals. From the plotting, by fusing the IMU measurements, the fusion results are smoother and more realistic.

In order to quantitatively evaluate the fused results using the uniform B-spline-based approach, we estimate the vehicle poses from the already optimized uniform B-splines at the time stamps, where we also have the exact vehicle pose measurements from our camera localization system. The difference between the poses estimated from the fused results using our approach and the poses from the camera localization system is evaluated in two parts: the translation part and the rotation part. Figure 4a illuminates the vehicle poses for a driving length of about 140 s, which has the translation part in the lower part and the rotation part in the upper part with the roll, pitch and yaw angles. The pose estimated from the visual localization system is highlighted with the blue color and the fusion results with the red color. As shown in Figure 4a, the pose differences between the fused results and the poses estimated from our visual localization system are very small. This evaluates our fusion approach in a macro way so that the low frequency pose measurements can be very well approximated in the fusion results. From the pose difference illustrated in Figure 4b, it is also easy to see that the estimated motion is much smoother and more meaningful for the driving maneuvers of a vehicle. This proves our fusion approach in a micro way; that the motion data between two visual localization measurements are continuous, accurate and reasonably approximated.

### 4.7. Point Cloud Accumulating

**Figure 5:**
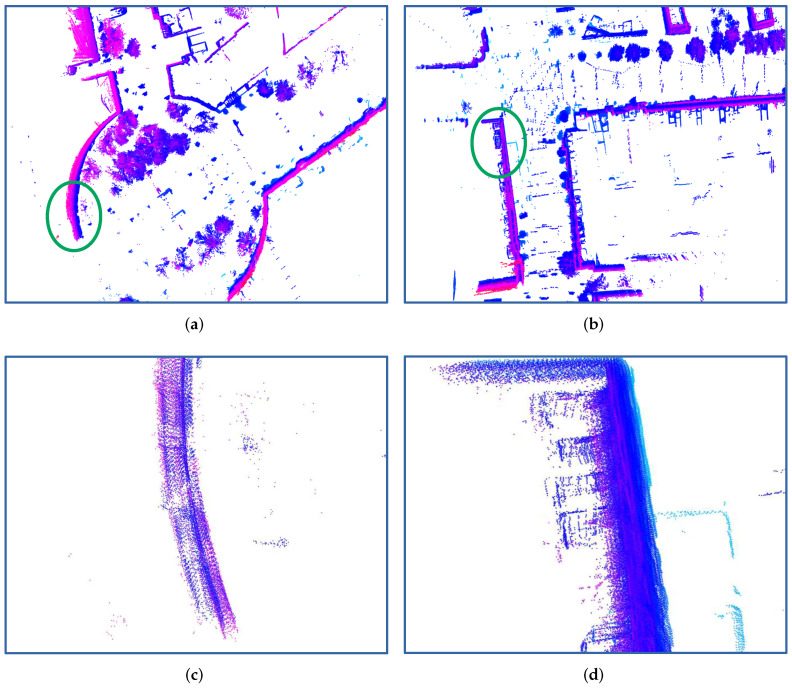

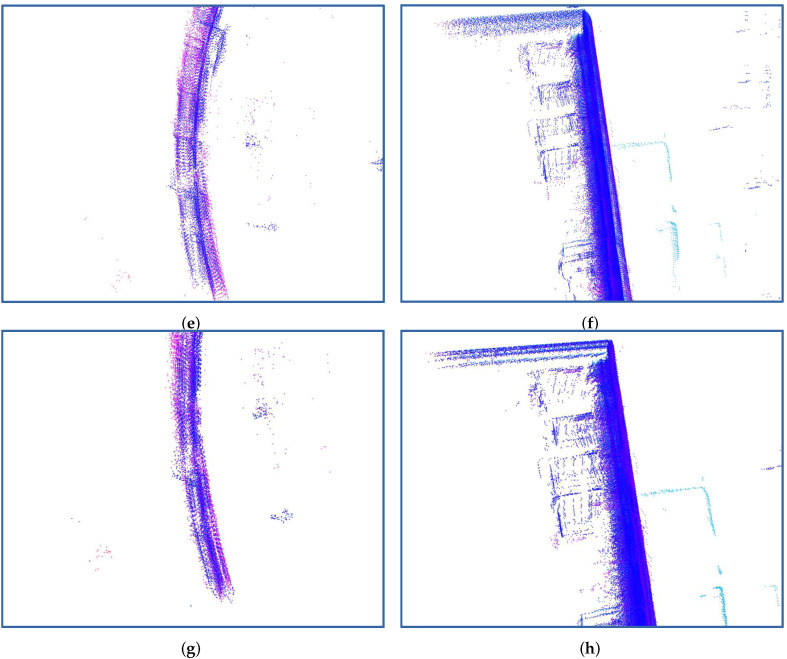
Point cloud accumulation results. (**a**,**b**) are two example scenarios. These two cropped areas are shown in detail in the lower part of images using the green ellipse; (**c**,**d**) present the accumulated point cloud using linear interpolation between two visual localization system measurements; (**e**,**f**) show the accumulated point cloud using the fusion results from the pose-graph-based approach; (**g**,**h**) visualize the accumulated point cloud using our fusion results.

After quantitatively evaluating the ego motion fusion results, to show the fusion results more clearly, we use the fusion results to accumulate the LiDAR point cloud packets coming with a high frequency of about 750 Hz. In Figure 5a,b, two accumulated point cloud examples from two different scenarios are shown. In Figure 5c,d, the raw LiDAR point cloud packets are accumulated using the linear interpolation between two poses estimated from our visual localization system. By applying the fused motion results using the pose-graph-based approach, the accumulated raw LiDAR point cloud packets are shown in Figure 5e,f. In comparison to only using the linear interpolation between visual localization poses, the pose-graph-based approach generates a smoother trajectory and the accumulated point cloud is also better motion-compensated. The results shown in Figure 5g,h use the fusion results from our approach, which are less blurry and more accurate in comparison with the data presented in Figure 5c–f. This shows that, by accumulating LiDAR point clouds with a very high frequency—which is also much higher than the estimated poses using the pose-graph-based fusion approach—we can achieve better motion-compensated results using the fused motion results with our approach. That is also one main contribution of this work: after fusing motion measurements using our uniform B-spline-based approach, we can time-continuously estimate the motion state without limitations on the time resolution.

## 5. Conclusions and Future Work

In this work, we propose a continuous fusion approach for motion measurements from different sensor systems with different types and frequencies. As the optimization base, we introduce uniform B-splines, which are well suited for this application because they are continuous, easily differentiable, computationally friendly and local support. To model rotation movements properly, we use the axis-angle rotation representation. It was revealed that this way of modelling the rotation can be combined easily with uniform B-splines and provides reasonable results. By applying different measurement models, we can fuse pose, velocity and acceleration measurements with different frequencies. As the fusion result, we achieve a trajectory, from which we can obtain the motion information at any time point. This benefits the processing steps after motion estimation for automated systems such as robotics, micro aerial and automated driving vehicles. For a time horizon of 10 s in our experiments, the optimization running on a single i7 CPU core takes less than 100 ms, which proves the suitability of our approach for realtime applications. In the future, we will introduce our fusion approach to LiDAR and visual or multi-modal SLAM systems, since they can benefit from the continuous estimation of motion for motion compensation and also from the asynchronous fusion of measurements from different sensor systems.
